# SACE_0012, a TetR-Family Transcriptional Regulator, Affects the Morphogenesis of *Saccharopolyspora erythraea*

**DOI:** 10.1007/s00284-013-0410-x

**Published:** 2013-06-23

**Authors:** Xiaojuan Yin, Xinqiang Xu, Hang Wu, Li Yuan, Xunduan Huang, Buchang Zhang

**Affiliations:** Institute of Health Sciences, School of Life Sciences, Anhui University, Jiu Long Road No. 111, Hefei, 230601 China

## Abstract

*Saccharopolyspora erythraea*, a mycelium-forming actinomycete, produces a clinically important antibiotic erythromycin. Extensive investigations have provided insights into erythromycin biosynthesis in *S. erythraea*, but knowledge of its morphogenesis remains limited. By gene inactivation and complementation strategies, the TetR-family transcriptional regulator SACE_0012 was identified to be a negative regulator of mycelium formation of *S. erythraea* A226. Detected by quantitative real-time PCR, the relative transcription of *SACE_7115,* the *amfC* homolog for an aerial mycelium formation protein, was dramatically increased in *SACE_0012* mutant, whereas erythromycin biosynthetic gene *eryA*, a pleiotropic regulatory gene *bldD*, and the genes *SACE_2141*, *SACE_6464*, *SACE_6040*, that are the homologs to the sporulation regulators WhiA, WhiB, WhiG, were not differentially expressed. *SACE_0012* disruption could not restore its defect of aerial development in *bldD* mutant, and also did not further accelerate the mycelium formation in the mutant of *SACE_7040* gene, that was previously identified to be a morphogenesis repressor. Furthermore, the transcriptional level of *SACE_0012* had not markedly changed in *bldD* and *SACE_7040* mutant over A226. Taken together, these results suggest that SACE_0012 is a negative regulator of *S. erythraea* morphogenesis by mainly increasing the transcription of *amfC gene*, independently of the BldD regulatory system.

**Electronic supplementary material:**

The online version of this article (doi:10.1007/s00284-013-0410-x) contains supplementary material, which is available to authorized users.

## Introduction

During its life cycle, the soil-inhabiting *Actinomycetes* undergoes a complex morphological differentiation to adapt to adverse environments [1]. Growth of *Actinomycetes* begins with spore germination and hyphal outgrowth, leading to the formation of a vegetative, or substrate mycelium. Sensing of nutrient deprivation stimulates reproductive growth resulting in the development of aerial hyphae and spore chains [2]. *Saccharopolyspora erythraea* could form the aerial hyphae, and produce erythromycin, which is a macrolide antibiotic with broad-spectrum antimicrobial activity. Extensive genetic and biochemical studies have provided detailed insights into the genes involved in erythromycin biosynthesis in *S. erythraea* [3, 4], yet its morphological differentiation remains poorly understood.

In recent years, the availability of the complete genome sequence of *S. erythraea* allowed a deeper exploration of the molecular processes controlling its morphogenesis [5]. Guided by in vitro and in vivo investigations, BldD (SACE_2077) was discovered to be a key developmental regulator in actinomycetes [1], controlling erythromycin biosynthesis and morphological differentiation in *S. erythraea* [6]. Furthermore, we identified a TetR-family transcriptional regulator (SACE_7040) involving in *S. erythraea* mycelium formation, and established genetic evidence for the crosstalk between SACE_7040 and BldD [7]. In this study, we have used gene inactivation, complementation, and transcriptional analysis to delineate the role of a new TetR-family regulator (SACE_0012) in the development differentiation of *S. erythraea*. Deletion of *SACE_0012* principally influences the transcription of a putative aerial mycelium formation gene *SACE_7115*, that is homologous to *amfC* of *Streptomyces*.

## Materials and Methods

### Strains and Growth Conditions

*Saccharopolyspora erythraea* A226 and its derivatives were incubated in TSB medium at 30 °C for DNA extraction, protoplast preparation, and in liquid fermentation medium R5 for analysis of erythromycin production. R3M agar medium was used for protoplast regeneration, phenotypic observations, and RNA extraction [7]. *Escherichia coli* DH5α was the host for plasmid construction [8]. *Bacillus subtilis* PUB110 was used for an inhibition test of erythromycin production in *S. erythraea.*

### Plasmid, DNA Isolation, and Manipulation

Plasmid pUCTSR [9] was a pUC18 derivative containing a 1.36 kb fragment of a thiostrepton resistance cassette (*tsr*) cloned into the *Bam*HI/*Sma*I sites. The *E. coli-* *S. erythraea* integrative shuttle expression vector pZMW [4, 10] was used for the gene complementation. DNA isolation and manipulation in *E. coli* and *S. erythraea* were carried out according to the standard methods [8, 11].

### Disruption of *SACE_0012* in *S. erythraea* A226, *bldD*, and *SACE_7040* Mutant

**Fig. 1:**
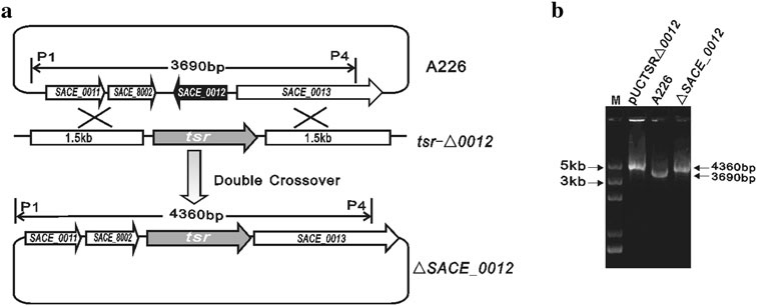
Inactivation of the TetR-family regulatory gene *SACE_0012*. **a** Schematic deletion of *SACE_0012* in *S. erythraea* A226. **b** Confirmation of *SACE_0012* deletion mutant by PCR analysis with the primer pair P1/P4. The size of 3.69 kb for the PCR-amplified bands was observed in wild-type A226, while a band of the size 4.36 kb was observed in mutant Δ*SACE_0012*, suggesting that the *SACE_0012* gene was completely deleted

Two 1.5 kb fragments flanking the *SACE_001*2 gene were amplified from genomic DNA of *S. erythraea* A226 by PCR using the primer pairs P1/P2 (5′-TGC GAA TTC CTC CTC GGC CGG TGA GCA GC-3′; 5′-GAT GGT ACC ATA CGA GCG GCC CCA ACC CGA AAG CCC-3′) and P3/P4 (5′-ATT TCT AGA ACA CGC CCG CCA CCG GCT TCG C-3′; 5′-ACC AAG CTT AAG GGC TCG ATC GAC TCC TGG CGG-3′). Then, the two DNA PCR products were inserted into the *Eco*RI/*Kpn*I and *Xba*I/*Hin*dIII sites of pUCTSR, respectively, yielding pUCTSRΔ*0012*. By linearized fragment homologous recombination [7], the *SACE_0012* gene was replaced with the thiostrepton resistance gene in the *S. erythraea* A226 chromosome, and the selected mutants were verified by PCR using the primers P1/P4 (Fig. 1a, b). Similarly, *SACE_0012* disruption was formed in the *bldD* and *SACE_7040* mutant strains.

### Genetic Complementation of the *SACE_0012* Mutant

For complementation, the *SACE_0012* gene was amplified by the primers P5 (5′-TAA CAT ATG TTG AAA ACG GCG TCA ATC CTC ATC CCG-3′) and P6 (5′-CGC GAT ATC TCA GCG ATC GGC GGT AGT CG-3′) from genomic DNA of *S. erythraea* A226, and was ligated into the *Nde*I/*Eco*RV sites of pZMW [9] to generate pZMW-*0012*. Then, pZMW-*0012* was introduced into *SACE_0012* mutant by PEG-mediated protoplast transformation, generating the complemented strain Δ*SACE_0012*/pZMW-*0012*.

### Quantitative Real-Time PCR (qRT-PCR)

The transcriptional levels of *eryA*, *bldD*, *SACE_0012*, and homologous genes of *whiA*, *whiB*, *whiG*, and *amfC* associated with morphogenesis in *Streptomyces* (Table S1) [12], were determined by qRT-PCR. Specific primers were designed as listed in Table S2. Total RNA was isolated from *S. erythraea* A226 and the mutants of *SACE_0012*, *bldD*, and *SACE_7040* after 2 or 4 days growth on R3M agar medium. Then, extracted RNA was treated with DNase I (Fermentas), and reverse transcription was accomplished using a cDNA synthesis kit (Fermentas). qRT-PCR reactions were performed on the Applied Biosystems StepOnePlus system with Maxima™ SYBR Green/ROX qPCR Master Mix (Fermentas). The *hrdB* gene encoding the major sigma factor in *S. erythraea* was used as an internal control, and relative quantification was evaluated using a comparative cycle threshold method as described by Livak and Schmittgen [13].

### Fermentation and Analysis

Wild-type strain A226, Δ*SACE_0012*, and Δ*bldD* were grown in 30 ml R5 liquid medium in 250 ml baffled flasks for 6 days at 30 °C. 5 μl fermentation supernatant from these cultures was added to LB agar plates, which was sprayed with an overnight culture of *B. subtilis* PUB110. The plates were incubated at 37 °C for 12 h, and the erythromycin production was estimated by scoring the growth-inhibition zones. Furthermore, erythromycin A produced by these cultures were quantitatively analyzed by high performance liquid chromatograph (HPLC) as described previously [14]. Erythromycin was isolated from the fermentation culture, and quantified by a standard curve [15].

## Results and Discussion

### Characterization of the *SACE_0012* Gene Deletion Mutant

Given a key role of the TetR-family regulator in morphological differentiation in actinomycetes [16], by gene inactivation and phenotype observation, we have identified several TetR-family regulators related to morphogenesis in *S. erythraea*, including the SACE_7040 gene previously reported [7] and the SACE_0012 gene currently studied. Bioinformatic analysis shows that the *SACE_0012* gene has a full-length of 690 bp (GenBank Accession No. NC-009142.114,813–115,502 nt) and is a member of the TetR-family regulators that consists of 229 amino acids with a molecular mass of 25 kDa. To investigate its function, *SACE_0012* was inactivated by replacing the 690 bp gene with a thiostrepton resistance cassette in *S. erythraea* A226 by the linearized fragment homologous recombination. A thiostrepton resistant mutant Δ*SACE_0012* was formed and confirmed by PCR analysis (Fig. 1a, b).

When grown on R3M medium, the mutant Δ*SACE_0012* formed aerial hypha earlier than original strain A226 in a three-day assay (Fig. 3b). When complemented with a cloned *SACE_0012* under the control of the PermE* constitutive promoter (pZMW-*0012*), the Δ*SACE_0012/*pZMW-*0012* strain had restored the timing of aerial mycelium on R3M agar medium (data not shown). After a longer cultivation to the sixth day, no significant phenotypic difference was observed between the wild-type strain A226, mutant Δ*SACE_0012*, and Δ*SACE_0012*/pZME-0012 (data not shown), revealing that *SACE_0012* was responsible for the early aerial hypha formation of *S. erythraea*. Moreover, Δ*SACE_0012* and A226 strains had comparable inhibition activity for *B. subtilis*, and produced similar amount of erythromycin A by HPLC analysis of fermentation products (Fig. S1A–B), confirming that SACE_0012 was specifically involved in the morphological differentiation of *S. erythraea*.

### Effect of *SACE_0012* Disruption on Transcription of the Genes for Morphogenesis and Erythromycin Biosynthesis

**Fig. 2:**
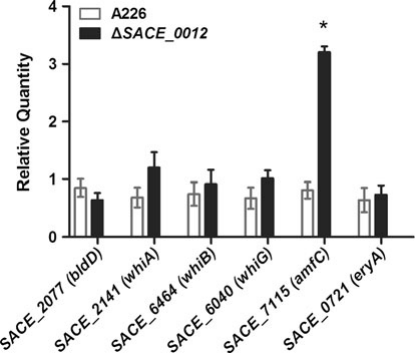
Transcriptional analysis of the genes for morphogenesis and erythromycin biosynthesis in the *SACE_0012* mutant. Mean values of three independent experiments are shown, with the standard deviation indicated by *error bars*. Statistical significance (*P < 0.05) compared to culture of wild-type strain A226

To test the effect of *SACE_0012* disruption on the expression of morphogenesis and erythromycin biosynthesis genes, we compared A226 and mutant Δ*SACE_0012* for the transcriptional change to sporulation genes (*whi* and *bldD*), an aerial mycelium formation gene *amfC* [17], and the erythromycin structure gene *eryA* (Table S1). The homologous genes to *whiA*, *whiB*, *whiG* involved in the regulation of sporulation in *Streptomyces* [18] (*SACE_2141*, *SACE_6464*, *SACE_6040*, respectively) were examined by qRT-PCR. *SACE_2141*, *SACE_6464*, *SACE_6040* transcriptions were slightly increased but not statistically different in mutant ∆*SACE_0012* over strain A226. *bldD* and *eryA* were also not differentially expressed. However, the transcriptional levels of the *amfC homolog SACE_7115* (Table S1), an aerial mycelium formation gene conserved presented in *Streptomyces* [17], were approximately 3.0-fold higher in the mutant ∆*SACE_0012* (Fig. 2).

### *SACE_0012* Disruption Failed to Restore the Defect in the Mycelium Formation of a *bldD* Mutant

**Fig. 3:**
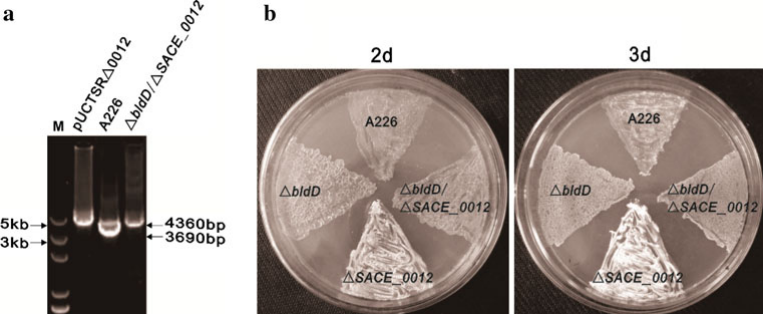
Confirmation and phenotype of the Δ*bldD/*Δ*SACE_0012* double mutant. **a** Confirmation of Δ*bldD*/Δ*SACE_0012* mutant by PCR analysis with the primer pair P1/P4. **b** Phenotype of the Δ*bldD*/Δ*SACE_0012* mutant

*bldD* is required for development differentiation in *S. erythraea* [1]. To examine the relationship of *SACE_0012* and *bldD*, *SACE_0012* was disrupted in the *bldD* mutant to form a double knockout mutant strain, Δ*bldD*/Δ*SACE_0012* (Fig. 3a). No significant phenotypic difference was observed between the mutant Δ*bldD*/Δ*SACE_0012* and Δ*bldD* (Fig. 3b), indicating that *SACE_0012* disruption could not restore the defect of aerial hyphae in *bldD* mutant. qRT-PCR analysis showed that *SACE_0012* transcriptions were slightly decreased but not obviously different in mutant ∆*bldD* (Fig. 5). These indicate that *SACE_0012*, although influencing morphological differentiation, seems to have no connection with the BldD regulatory system.

### *SACE_0012* Disruption Did Not Further Accelerate the Mycelium Formation of *SACE_7040* Mutant

**Fig. 4:**
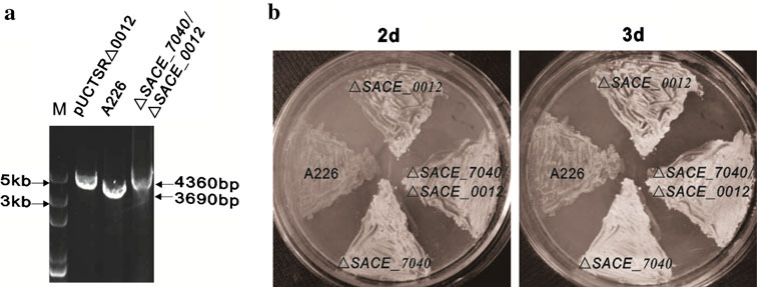
Confirmation and phenotype of the Δ*SACE_7040*/Δ*SACE_0012* double mutant. **a** Confirmation of Δ*SACE_7040*/Δ*SACE_0012* mutant by PCR analysis with the primer pair P1/P4. **b** Phenotype of the Δ*SACE_7040*/Δ*SACE_0012* mutant

**Fig. 5:**
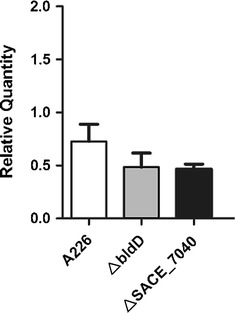
Relative transcriptional levels of *SACE_0012* in the deletion mutants of *bldD* and *SACE_7040* over A226. Mean values of three independent experiments are shown, with the standard deviation indicated by *error bars*

We identified a TetR-family regulator SACE_7040 negatively involving in the morphological differentiation of *S. erythraea*, in which an interplay with the *bldD* gene was previously established [7]. Therefore, we inactivated the *SACE_0012* gene in the *SACE_7040* mutant to study combined effect upon the morphogenesis (Fig. 4a). It appears that the mycelium formation of *SACE_7040* mutant was earlier than *SACE_0012* mutant, but no obvious change of aerial mycelia was detected in the double knockout mutant Δ*SACE_7040*/Δ*SACE_0012* relative to the *SACE_7040* mutant (Fig. 4b). Likewise, *SACE_0012* transcriptions were slightly decreased but not statistically different in mutant ∆*SACE_7040* (Fig. 5).

In conclusion, these results indicated that compared with original strain A226, aerial hypha formation initiates earlier in *SACE_0012* mutant. The likely cause of an early aerial hypha formation is the higher transcriptional level of *amfC*. Previous genetic evidences revealed that *amfC* positively controlled aerial mycelium formation in *Streptomyces coelicolor* and *Streptomyces griseus*, and was distributed widely in this genus [17], implying that *amfC* might be under the control of *SACE_0012* to affect the early aerial hypha formation of *S. erythraea*. In addition, we found that *SACE_0012* disruption could not restore the defect of aerial development in a *bldD* mutant, and also could not further accelerate the mycelium formation in a mutant of *SACE_7040* gene. Further qRT-PCR analysis showed that *SACE_0012* transcriptions were slightly decreased but not obviously different in mutant ∆*bldD and* ∆*SACE_7040* relative to A226. Thus, *SACE_0012* was likely independent of the BldD regulatory system for controlling *S. erythraea* morphogenesis, distinct from the TetR-family regulator *SACE_7040* previously reported [7].

With structural and sequence conserved analysis [19], homologous of *SACE_0012* are predominantly distributed in rare actinomycetes, such as *Amir_6428* from *Actinosynnema mirum* (identities 50 %), *BN6_77090* from *Saccharothrix espanaensis* (identities 50 %), *AMED_0889* from *Amycolatopsis mediterranei* (identities 48 %), etc. (Fig. S3). However, functional analysis of the TetR-family regulator has been never reported, signifying a new regulatory mechanism for mycelial formation in actinomycetes, such as how it works with its ligand and target [10]. Therefore, these findings provide novel insights into *S. erythraea* developmental biology. In the future, more detailed regulatory mechanism of the *SACE_0012* gene will likely be valuable to deepening the understanding of the modulation of *S. erythraea* morphogenesis.

## Electronic supplementary material

Below is the link to the electronic supplementary material.

Supplementary material 1 (DOC 703 kb)
