# Supplementary material for: SACE_0012, a TetR-Family Transcriptional Regulator, Affects the Morphogenesis of Saccharopolyspora erythraea
Source: Curr Microbiol. 2013 Jun 23;67(6):647–51. doi: 10.1007/s00284-013-0410-x (PMC3825060; doi:10.1007/s00284-013-0410-x)
Supplement: Supplementary file 1 — Supplementary material 1 (DOC 703 kb) [file 284_2013_410_MOESM1_ESM.doc]

**Supplementary information**


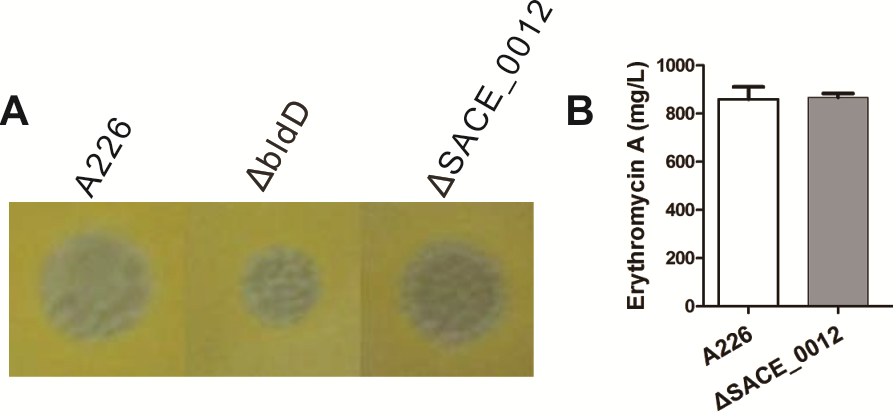


**Fig S1. Effect of *SACE_0012* deletion on erythromycin production in *Sac. erythraea* A226.** (A) Inhibition tests of wild-type strain A226 and *SACE_0012* deletion mutant Δ*SACE_0012* against *Bacillus subtilis*; (B) Erythromycin A production by A226 and Δ*SACE_0012,* in liquid fermentation medium R5*.* Mean values of three independent experiments with SD are indicated by error bars.

**
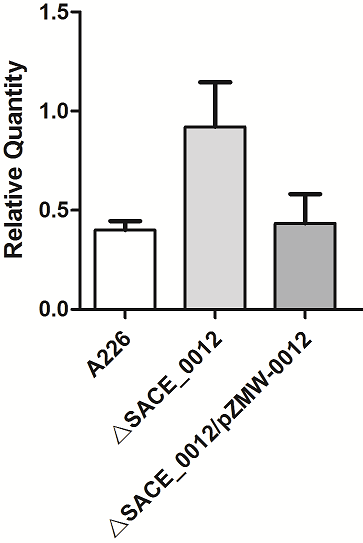
**

**Fig S2. Transcriptional analysis of the *amfC* homolog *SACE_7115* in wide-type strain A226, *SACE_0012* mutant and its complemented strain.** Mean values of three independent experiments are shown, with the standard deviation indicated by error bars.

**
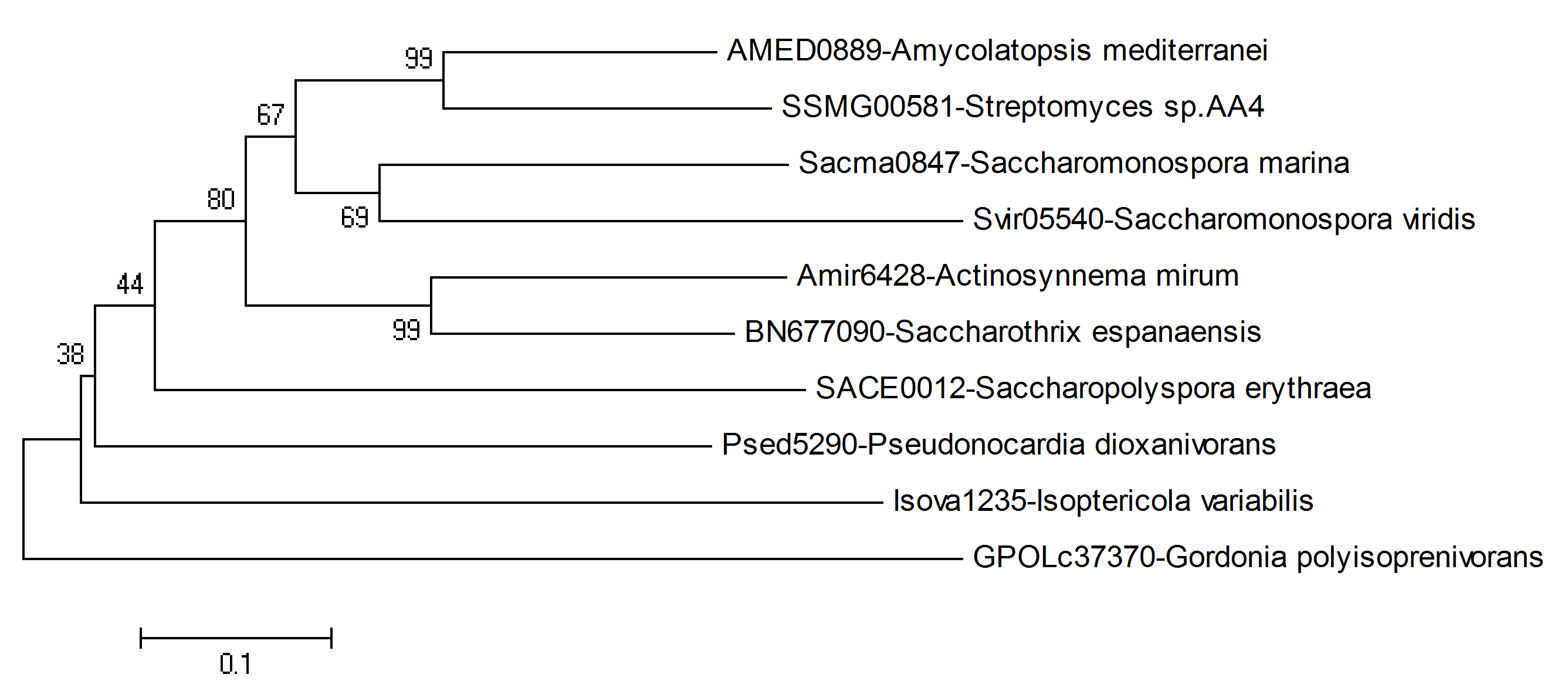
**

**Fig S3. Phylogenetic tree of SACE_0012 homologues by the neighbor-joining method.** The number at each node indicates the percentage of 1000 bootstrap replications.

**Table S1. Morphogenesis genes in *Streptomyces coelicolor* and its homologs in *Saccaropolyspora erythraea***

| ***S. coelicolor*** | ***Sac. erythraea*** | **Homology (%)*** | **Gene product** |
| --- | --- | --- | --- |
| *whiA (SCO1950)* | *whiA (SACE_2141)* | 75 | sporulation regulatory protein |
| *whiB (SCO3034)* | *whiB (SACE_6464)* | 79 | WhiB-family transcriptional regulator |
| *whiG (SCO5621)* | *whiG (SACE_6040)* | 56 | RNA polymerase sigma factor |
| *bldD (SCO1489)* | *bldD (SACE_2077)* | 77 | transcriptional regulator |
| *amfC (SCO4184)* | *amfC (SACE_7115)* | 41 | aerial mycelium formation protein |

*Amino acid sequence identity between *S. coelicolor* and *Sac. erythraea*.

**Table S2. The primers used for qRT-PCR in this study**

| **Primer** | **Sequence (5'-3')** |
| --- | --- |
| *hrdB*-F | GGTCACGCCGTAGACCTGGC |
| *hrdB*-R | CGGTGTCGTTCACGCTGCTG |
| *bldD*-F | ACGACTTTGGTGGCGGGCTC |
| *bldD*-R | CCGTGGTCGTCGGGTCCTAT |
| *whiA*-F | GCACGTCGGACTGGTGGC |
| *whiA*-R | TCTGAGGTGTCGTCGCTGCT |
| *whiB*-F | GAGGGCGTATTCCAGGCATT |
| *whiB*-R | GGGGAGGAAGAACAGGAATGG |
| *whiG*-F | ACTGGATCAGGTCCGAAACC |
| *whiG*-R | TCGTGGCTCTGTGGAAGGC |
| *amfC*-F | TCCTCGGCGGTGTAGTGG |
| *amfC*-R | CGGGCGGTACCAGACGAT |
| *eryA*-F | *CCGCTGATGCCGAACGAC* |
| *eryA*-R | *CACCCTTCCCCGCACTCTG* |
| *SACE_0012*-F | TGCCACCGAACAGCGAGT |
| *SACE_0012*-R | AGGGTCCACGACGACAAGC |
